# Giving It Our Best Shot? Human Papillomavirus and Hepatitis B Virus Immunization Among Refugees, Massachusetts, 2011–2013

**DOI:** 10.5888/pcd14.160442

**Published:** 2017-06-22

**Authors:** Rachel Stein Berman, Laura Smock, Megan H. Bair-Merritt, Jennifer Cochran, Paul L. Geltman

**Affiliations:** 1 Department of General Pediatrics, Boston University School of Medicine/Boston Medical Center, Boston, Massachusetts; 2 Refugee and Immigrant Health Program, Massachusetts Department of Public Health, Boston, Massachusetts

## Abstract

**Introduction:**

The receipt rate of hepatitis B virus vaccine among adolescents in the United States is high, while the receipt rate of human papillomavirus vaccine is low. Rates have not been closely studied among refugees, whose home countries have high rates of disease caused by these viruses.

**Methods:**

We examined human papillomavirus and hepatitis B virus immunization rates among 2,269 refugees aged 9 to 26 years who resettled in Massachusetts from 2011 through 2013. This was a secondary analysis of data from their medical screenings. We used binary logistic regression to assess characteristics associated with immunization and bivariate analyses to compare refugee immunization rates with those of the general US population.

**Results:**

Forty-five percent of US adolescents aged 13 to 17 years received 1 dose of human papillomavirus vaccine, compared with 68% of similarly aged refugees. Males (adjusted odds ratio [aOR], 0.62; 95% confidence interval [CI], 0.52–0.74), refugees older than 13 years (aOR, 0.74; 95% CI, 0.60–0.93), and refugees not from Sub-Saharan Africa (aOR, 0.74; 95% CI, 0.59–0.92) were less likely to receive human papillomavirus vaccine, while arrivals in 2012 through 2013 were more likely (aOR, 1.6; 95% CI, 1.3–1.9) than those arriving in 2011. Refugees older than 13 years were less likely to receive 2 doses of hepatitis B virus vaccine (aOR, 0.49; 95% CI, 0.37–0.63) than older refugees.

**Conclusion:**

Specialized post-arrival health assessment may improve refugees’ immunization rates.

## Introduction

Since 2009, approximately 70,000 refugees each year resettle in the United States. Of these, 30% to 40% are children (1). Refugee children immigrate with substantial preventable health problems, including infectious diseases, elevated lead levels, dental problems, and poor nutrition (2–4), and have low overall immunization rates (5). Language and cultural differences, poverty, trauma, and lack of insurance are barriers to care once resettled (4,6).

Cervical cancer and sequelae of hepatitis B infection are more common in refugees’ countries of origin than in the United States. These conditions are caused by human papillomavirus (HPV) and hepatitis B virus (HBV), respectively, and each condition is preventable with a 3-dose vaccine series (7–10). Although HBV immunization rates are high in the United States, with 93% of those aged 13 to 17 years receiving the series in 2013, only 45% of US adolescents initiated the newer HPV vaccine series in 2013. For both vaccines, disparities in coverage exist by race and ethnicity (11). Patients’ and providers’ attitudes and beliefs, parent education, insurance status, and having a reliable source of health care can influence receipt of immunization (12).

Although studies have explored disparities in immunization by race and ethnicity, they have not examined data on immunization among refugees, who differ from other immigrants by countries of origin, medical conditions, experiences with the health care system (post-arrival medical screening), and trauma. The objective of this study was to analyze HPV and HBV immunization rates among refugee children resettled in Massachusetts, to examine sociodemographic factors associated with immunization among refugees, and to compare immunization rates among refugees with those of the general US population. We anticipated that our findings would facilitate culturally relevant immunization and preventive health services for refugees.

## Methods

### Study design, participants, and data collection

This study entailed a secondary analysis of data from a cohort of refugees resettled in Massachusetts from 2011 through 2013. Nearly all refugees resettled in Massachusetts are screened through the Refugee Health Assessment Program (RHAP) of the Massachusetts Department of Public Health (MDPH). RHAP is a contracted network of 12 private clinic sites and community health centers that provides health screening and immunization according to a clinical protocol established by MDPH for refugees resettled in Massachusetts. RHAP serves as initial access to primary care services, with each new refugee having 2 visits within 90 days of arrival (4). During an RHAP visit, refugees undergo vision, hearing, behavioral health, and dental screening; have laboratory samples drawn to screen for hepatitis B, parasites, human immunodeficiency virus, anemia, vitamin D deficiency, abnormal urine chemistry, and exposure to tuberculosis; receive immunizations per guidelines of the Centers for Disease Control and Prevention (CDC); and are provided information about how to access health care in Massachusetts (4,13).

The study sample consisted of all refugees aged 9 to 26 years resettled in Massachusetts from 2011 through 2013 with documented medical screening through RHAP. Data were extracted from the Massachusetts Virtual Epidemiologic Network (MAVEN), which is the MDPH’s web-based surveillance and case management system (14). MAVEN includes demographic and clinical data derived from health screenings overseas and shortly after arrival in Massachusetts. Examples of the clinical data include anthropometrics and vital signs, health conditions diagnosed overseas and domestically, dates of immunizations, screening test results, and medications prescribed pre-arrival and post-arrival (15).

### Measures

The primary outcomes of interest were receipt of 1 dose of HPV vaccine and 2 doses of HBV vaccine pre-arrival and post-arrival. We chose to look at 1 dose of HPV vaccine and 2 doses of HBV vaccine because timing of RHAP visits does not allow for completion of either series. Refugees often receive their first dose of HBV vaccine overseas, but HPV vaccine is generally not available in the countries from which refugees in Massachusetts migrate (16). Therefore, the expectation was that the initial dose of HPV vaccine would be given during an RHAP visit. We extracted data for the following predictor variables: date of arrival, age, nationality, sex, race, presence of acute or chronic medical conditions, HBV surface antigen status, anti-HBV surface antibody status, and total number of vaccines received pre-arrival and post-arrival (not aggregated by vaccine type). Nationality was categorized by world region of origin, based on US Department of State regional categories. Three countries — Somalia, Iraq, and Bhutan — were evaluated separately from their world region categories because they had a sufficient number of refugee arrivals to warrant individual analyses. We examined data on acute and chronic medical conditions to control for clinicians’ decisions to postpone vaccination because of illness. We also extracted data for the covariates of dates of RHAP clinic visits and the RHAP clinic site.

We converted 2 continuous variables into categorical variables for facilitation of analysis. We converted age to a dichotomous variable — 9 to 12 years or 13 to 26 years — to separate refugees who had been vaccinated per CDC guidelines at age 11 or 12 years from those who had received the vaccine later. We also converted year of arrival to a dichotomous variable — arriving in 2011 or arriving in 2012 through 2013 — to examine whether vaccination rates changed when RHAP began emphasizing the administration of new vaccines, such as the meningococcal conjugate vaccine and the HPV vaccine, in 2012.

### Analyses

We calculated means and standard deviations for all continuous variables and frequencies for all categorical variables. We then performed bivariate analyses (*t* test and χ^2^ analyses, as appropriate) to assess associations between HPV and HBV immunization and the variables listed above. Significance was based on a *P* value of less than .05. On the basis of these results, we conducted logistic regression analyses to assess factors associated with vaccine receipt. The variables included in the logistic regression for HPV immunization were age group, sex, RHAP site, arrival year, and world region of origin. The world region of origin variable was dichotomized for the logistic regression into 1) Sub-Saharan Africa, excluding Somalia, and 2) other regions. These were dichotomized because this region had the highest rate of HPV immunization among the regions studied. The variables included in the logistic regression for HBV immunization were age group, RHAP site, HBV surface antigen status, and anti-HBV surface antibody status. Lastly, we compared HPV immunization rates among refugees aged 13 to 17 years with rates in the general US population and the general Massachusetts population of the same age, based on National Immunization Survey (NIS) data, using χ^2^ analyses. We used SAS version 9.3 (SAS Institute, Inc) to conduct our statistical analyses. This study was approved by the authors’ institutional review boards and was monitored by MDPH’s Institutional Review Board.

## Results

From 2011 through 2013, 2,269 refugees aged 9 to 26 years were resettled in Massachusetts. Predominant nationalities were Iraqi (25%), Bhutanese (24%), and Somali (11%) (Table 1). Refugees received a mean of 9.3 individual vaccines (range, 0 to 31), administered both overseas and at RHAP visits. No single variable had greater than 3% of values missing, and over 95% of refugees included in the analysis had complete data.

**Table 1:** Refugee Characteristics (N = 2,269), Massachusetts Department of Public Health, 2011–2013

Characteristic	N (%)
**Age, y**	
9–12	431 (19)
13–26	1,838 (81)
**Arrival year**	
2011	696 (31)
2012	821 (36)
2013	752 (33)
**Sex^a^**	
Female	1,040 (46)
Male	1,228 (54)
**Race^b^**	
White	679 (30)
Black	803 (35)
Asian	765 (34)
Other	12 (0.5)
Unknown	10 (0.4)
**Region^c^**	
Sub-Saharan Africa, excluding Somalia	481 (21)
Somalia	255 (11)
Near East, excluding Iraq	24 (1)
Iraq	571 (25)
South and Central Asia, excluding Bhutan	29 (1)
Bhutan	538 (24)
East Asia and Pacific	214 (9)
Europe and Eurasia	64 (3)
Western Hemisphere	93 (4)

a One refugee had missing data for sex.

b Ten refugees had missing data for race.

c World regions are the regions used by the US Department of State. Three countries — Somalia, Iraq, and Bhutan — were evaluated separately from their world region categories because they had a sufficient number of refugee arrivals to warrant individual analyses.

Fifty-six percent of all refugees aged 9 to 26 years received 1 dose of HPV vaccine, either pre-arrival or post-arrival. Among all refugees, those who received 1 dose of HPV vaccine received 3.3 more vaccines than refugees who did not receive HPV vaccine (*P* < .001). Males were less likely than females to receive HPV vaccine (adjusted odds ratio [AOR], 0.62; 95% confidence interval [CI]. 0.52–0.74). Refugees aged 13 to 26 years were less likely to receive HPV vaccine than refugees aged 9 to 12 years (AOR, 0.74; 95% CI, 0.60–0.93). Refugees who were not from Sub-Saharan Africa, excluding Somalia, were less likely than refugees from this region to receive HPV vaccine (AOR, 0.74; 95% CI, 0.59–0.92). Arrivals in 2012 through 2013 were more likely to receive HPV vaccine than refugees who arrived in 2011 (AOR, 1.6; 95% CI, 1.3–1.9). The only relevant acute or chronic medical conditions present among this sample of refugees were acute upper respiratory infections and ear abnormalities and infections, neither of which was found to be significantly associated with receipt of 1 dose of HPV vaccine. Rates of HPV immunization varied significantly by RHAP clinic site.

Rates of HBV immunization varied: 91% of refugees received at least 1 dose, 63% received 2 doses, and 10% received 3 doses. Timing of RHAP visits, typically 1 month apart, generally does not permit administration of a third dose, which should be 2 months after the second dose and 6 months after the first dose. Refugees aged 13 to 26 years were less likely to receive 2 doses of HBV vaccine than refugees 9 to 12 years old (AOR, 0.49; 95% CI, 0.37–0.63) (Table 2). Refugees with positive HBV surface antigen or antibody were less likely to receive 2 doses than those with negative antigen (AOR, 0.13; 95% CI, 0.07–0.24) or antibody (AOR, 0.42; 95% CI, 0.34–0.52) status. Nationality, year of arrival, sex, and presence of acute upper respiratory infections or ear abnormalities and infections were not significantly associated with receipt of HBV vaccine. Furthermore, we found no significant difference in receipt of 2 doses of HBV vaccine among refugees who had received 1 or more doses overseas or both doses during RHAP visits.

**Table 2:** Refugee Characteristics Predictive of Vaccine Receipt (N = 2,269), Massachusetts Department of Public Health, 2011–2013

Characteristic	Human Papillomavirus	Hepatitis B Virus
**Age, y (reference: 9–12 y), adjusted odds ratio (95% confidence interval)**		
13–26 years	0.74 (0.60–0.93)	0.49 (0.37–0.63)
**Sex (reference: female), adjusted odds ratio (95% confidence interval)**		
Male	0.62 (0.52–0.74)	1.1 (0.94–1.3)
**Arrival year (reference: 2011), adjusted odds ratio (95% confidence interval)**		
2012–2013	1.6 (1.3–1.9)	0.87 (0.70–1.1)
**Hepatitis B virus surface antigen (reference: negative), adjusted odds ratio (95% confidence interval)**		
Positive	Does not apply	0.13 (0.07–0.24)
**Hepatitis B virus surface antibody (reference: negative), adjusted odds ratio (95% confidence interval)**		
Positive	Does not apply	0.42 (0.34–0.52)
**Region, % (n) [*P* value^a^]**		
Sub-Saharan Africa, excluding Somalia (N = 481)	63 (302) [Reference]	68 (326) [Reference]
Somalia (N = 255)	51 (129) [.001]	50 (127) [<.001]
Near East, excluding Iraq (N = 24)	29 (7) [.002]	42 (10) [.01]
Iraq (N = 571)	61 (350) [.62]	59 (338) [.004]
South and Central Asia, excluding Bhutan (N = 29)	41 (12) [.03]	57 (17) [.31]
Bhutan (N = 538)	56 (301) [.03]	68 (364) [.97]
East Asia and Pacific (N = 214)	56 (119) [.07]	66 (141) [.49]
Europe and Eurasia (N = 64)	23 (15) [<.001]	50 (32) [.006]
Western Hemisphere (N = 93)	48 (45) [.009]	71 (66) [.61]

a χ^2^ analysis.

Refugee adolescents aged 13 to 17 years resettled in Massachusetts had higher rates of receipt of 1 dose of HPV vaccine than adolescents in the general US population. Per NIS data for the US population, in 2013, 45% of those aged 13 to 17 years received 1 dose of HPV vaccine compared with 68% of similarly aged refugees (odds ratio [OR], 2.5; 95% CI, 2.1–3.0). NIS data for the US population also showed that 57% of females and 35% of males aged 13 to 17 years received 1 dose; data for Massachusetts showed that 62% of females and 53% of males in this age group received 1 dose. RHAP data for this age group showed that 68% of female refugees (compared with US females: OR, 1.6; 95% CI, 1.2–2.1) and 68% of male refugees (compared with US males: OR, 3.9; 95% CI, 3.1–5.0) received 1 dose (11). Data were not available to calculate ORs for Massachusetts.

## Discussion

HBV immunization rates among refugees were higher than HPV immunization rates, with 91% of refugees receiving 1 dose and 63% of refugees receiving 2 doses of HBV vaccine. Refugees aged 13 to 26 years and refugees with positive HBV surface antigen had significantly lower rates of receipt of 2 doses of HBV vaccine than younger refugees and refugees with negative HBV surface antigen status. We had a similar finding for refugees who were positive for anti-HBV surface antibodies. This latter finding suggests that clinicians did not finish incomplete HBV vaccine series started overseas if the titer was positive. Lack of comparable data precluded comparison of HBV immunization rates among refugees with national and state data.

Our finding that female refugees were significantly more likely than male refugees to receive 1 dose of HPV vaccine is consistent with trends in the general population. These results are likely explained by the later recommendation for male vaccination. The CDC’s Advisory Committee on Immunization Practices (ACIP) first recommended HPV vaccine in 2006 for females aged 11 to 12 years, with catch-up vaccination for women up to age 26. The ACIP began recommending HPV vaccine for males aged 11 to 12 years in 2011, with catch-up vaccination for men up to age 21 (17). These results also may be explained by the general population’s stronger association of HPV with cervical cancer than with cancers that affect men (18).

The higher rates of HBV immunization compared with those for HPV immunization among refugees may be explained by the practice of administering 1 or more doses of HBV vaccine to refugees before their departure to the United States and the unavailability of HPV vaccine in their countries of origin. Although HBV vaccine is widely available and used around the world, the HPV vaccine is available and recommended mainly in countries in Europe and North and South America; however, most refugees resettled in Massachusetts did not originate in these regions (16).

The higher rates of HPV immunization among refugee adolescents compared with the general US adolescent population was unexpected, particularly given disparities in health and access to care by race and ethnicity as well as language and cultural barriers. Furthermore, studies have identified a lack of knowledge and awareness about HPV as well as certain beliefs about the vaccine among specific ethnic and racial groups that deter caregivers from initiating HPV vaccination for their children. Two studies of Latino parents showed that lack of knowledge about HPV, concerns about side effects, out-of-pocket costs, and concerns that the vaccine promotes sexual activity discourage parents from immunizing their children against HPV (19,20). Furthermore, among Cambodian American women, major barriers to HPV vaccination were lack of information and concerns about safety, cost, and effectiveness, while primary factors facilitating vaccination were provider recommendation and belief in the importance of disease prevention (21). When looking across racial and ethnic groups, one study among Latin American, Asian American, African American, and white women showed that fewer first-generation immigrants had heard of HPV and had lower knowledge of HPV compared with those who were second- and third-generation immigrants (22). The findings of our study suggest that the refugee populations we studied may differ from these immigrant populations. As the literature suggests, many caregivers hesitate to administer HPV vaccine to their children for cultural and religious reasons. It is possible that refugees are not aware of these hesitations and therefore do not object to administering the HPV vaccine to their children.

CDC has made several recommendations to address these hesitations. One factor that encourages caregivers to immunize their children against HPV is clinician recommendation (21–25), which may be particularly important for immigrants and refugees with limited access to health information (26). CDC therefore urges clinicians to encourage HPV vaccination and proposes doing so by making it routine. CDC suggests that clinicians tell caregivers “your child needs these vaccines today” and then list all of the vaccines, including HPV, for which the child is eligible by age (27). CDC also recommends that when discussing HPV vaccine with caregivers, providers highlight that the vaccine is a means to prevent cancer, is safe and effective, is for males and females, and should be given to children before possible virus exposure (28).

Applying these prior studies’ findings and CDC guidelines, the HPV and HBV vaccines are recommended by clinicians and made normative at every RHAP visit because they are part of the program’s clinical protocol requiring clinicians to follow ACIP guidelines. Given that the ACIP immunization schedule includes HPV vaccination for both males and females at 11 or 12 years old, RHAP clinicians may have been more likely to administer HPV vaccine to refugees of both sexes in this age group than to older refugees, explaining the higher HPV and HBV immunization rates of younger refugees and the equal rates of HPV immunization among male and female refugees aged 13 to 17 years.

The higher rates of HPV immunization among refugees resettled later in our study period were not surprising. RHAP began emphasizing the administration of HPV vaccine in late 2012 through annual oversight meetings with clinical staff at all RHAP sites, through 2 conference calls with audiovisual materials to support updates in the RHAP clinical protocol, and through email reminders. The higher HPV immunization rates later in our study period may also be explained by a shift in cultural norms and greater acceptance of the HPV vaccine in the United States as a whole, as rates of HPV immunization increased significantly from 2012 to 2013 for adolescent females and from 2011 to 2012 as well as from 2012 to 2013 for adolescent males (29).

Our study thus suggests that the use of a clinical immunization protocol by clinics specifically contracted by MDPH to perform health assessment of newly arrived refugees greatly increases vaccine receipt for refugees. Given the efficacy of the HPV and HBV vaccines in preventing cancer and liver disease, the adoption of provider recommendation and standardization of these vaccines in practice across the United States has positive public health implications in preventing illness and death from these viruses (9,28).

This study has several limitations. First, immunization rates of refugees resettled only in Massachusetts were assessed, and our data may not be generalizable to refugee populations in other states. Different refugee populations are resettled in different states (30), and states have different policies and procedures for resettlement (4). Immunization rates also vary by state, and the general adolescent population in Massachusetts has higher HPV immunization rates than the general adolescent population in other states (11). Second, because most refugees have only 2 RHAP visits, the data did not allow review of series completion for both the HPV and HBV vaccines. Third, the study was a secondary analysis of an existing data set that did not address why refugees or their caregivers did or did not choose to initiate HPV vaccination, how providers discussed the vaccine, or to what extent RHAP clinic sites standardized HPV immunization. Furthermore, research is needed to evaluate refugee immunization rates in other states, to determine refugees’ full-series completion rates for both vaccines, and to explore reasons for the different rates, such as refugees’ attitudes and beliefs, provider characteristics, and clinic protocols.

With already high rates of HBV and other immunizations, both among refugees and the general population in the United States, efforts should be directed to promote use of HPV vaccine among refugees and others. Through a collaborative campaign to promote HPV vaccination, both through universal clinician recommendation and through standardization of HPV vaccination, it is hoped that rates of HPV immunization of refugees and other children will continue to rise.
